# Lacan and Adolescence: The Contemporary Clinic of the “Sexual Non-rapport” and Pornography

**DOI:** 10.3389/fpsyg.2017.02299

**Published:** 2018-02-06

**Authors:** Olivier Ouvry

**Affiliations:** UTRPP Laboratory (EA 4403), Department of Psychology, University of Paris-Sorbonne-Cité, Paris, France

**Keywords:** conspiracy thought, pornography, the adolescent boy, adolescence, the Real of puberty, the other jouissance, sexual non-rapport

## Abstract

This article explores two clinical phenomena—pornography and conspiracy thinking—that are highly relevant today and can be observed specifically among adolescent boys in the early stages of post-puberty: conspiracy thinking and the viewing of pornographic videos. It shows that the Lacanian concepts of the Real (of puberty) and the sexual non-rapport help us understand the psychopathological aspects of these two phenomena. Watching pornographic material becomes equivalent to a conspiracy theory about the sexual non-rapport; both in fact deny the effect of what puberty introduces as radically new.

## Introduction

In our clinical experience, the two phenomena, which we are going to handle—pornography and conspiracy thinking^1^—can mostly be observed among post-pubertal boys. The already high and apparently growing occurrence of these manifestations seems troubling and often gives rise to highly controversial views^2^. What does this tell us? How do we understand these phenomena and deal with them? And should we indeed be worried or should we dismiss them as banal, given how widespread they currently are?

Our hypothesis is that these two phenomena, observed at teenagers not presenting known psychological disorders, are in fact interlinked through an underlying mechanism, which has to do with the dread of the other of the Other sex^3^. However, to arrive at this conclusion, we must first make a few preliminary remarks.

## Background

We can begin with the fact that these phenomena are observed mainly among boys. Hence, there is a gender division. We know that Freud saw the period of infantile sexuality as radically different for girls versus boys, and even located their Oedipal entrance and exit points as mirror images of one another. However, what happens to this difference in puberty?

For the boy, the end of the Oedipal period is marked by castration, which is experienced as a possibility when the reality principle of the existence of two sexes sets in, i.e., a reality that contradicts the infantile sexual theories that had previously kept it at bay. Anatomically, we are (born as) girls or boys, rather than becoming either sex (Beauvoir, 1949). This realization makes the imaginary register of the infantile sexual theories obsolete and replaces it with a logical order guaranteed by the father as the bearer of the organ that gives meaning to what makes difference in language (the Symbolic and the Real). It is through the articulation of the reality of the difference of the sexes to the lack in Other that the function of the phallus (the signifier of the lack in the Other one) find its validation in language.

For the little boy, the end of the Oedipus complex is therefore characterized by a change in the reference framework. This is why Freud speaks about the boy's *dissolution of the Oedipus complex* (Freud, 1924) rather than simply its repression. In this new world, everyone has their place as either a man or a woman—and everyone is asked to position themselves clearly.

However, the little girl does not follow the same path. When she sees the difference between the sexes, she does not prevaricate and decides that she does not have a penis (Freud, 1925). Prior to extending this destiny to all other girls, including her mother—and hence realizing how things are—during the pre-Oedipal stage the girl is in exactly the same position as the boy, as emphasized by Freud: they share the same sexual object (the mother) and the same erogenous zone (the clitoris—equated with the penis). The shift from the mother to the father as the sexual object is therefore produced via castration, introducing the Oedipal complex, which represents the first difference with the boy's trajectory^4^ (the girl does not keep the mother as her sexual object). From the father, she expects to receive the penis she does not have (like her mother), and which she converts, through a symbolic “equation” (Freud, 1925), into having a child from him.

This means that the girl enters the Oedipal stage precisely through the castration complex—i.e., by taking the reality of sexual difference into account—contrary to the boy, to whom the same realization provides a way of exiting and dissolving the Oedipus complex. For the girl, not only is there no defined exit from the Oedipus complex (she is not concerned by the threat of castration, see Freud), but also, importantly, her new logical position is that of seducing the father, who is a man, in order to unconsciously fulfill her wish of having a child with him.

This position of seduction, which the little girl acquires already as a child, has to be related to what Freud says in *Narcissism: An Introduction*, when he describes the “fundamental differences” between the male and female approach to love, in his text obviously speaking about the situation post-puberty. He writes:

A comparison of the male and female sexes then shows that there are fundamental differences between them in respect of their type of object-choice, although these differences are of course not universal. Complete object-love of the attachment type is, properly speaking, characteristic of the male. […] A different course is followed in the type of female most frequently met with, which is probably the purest and truest one. With the onset of puberty, the maturing of the female sexual organs, which up till then have been in a condition of latency, seems to bring about an intensification of the original narcissism, and this is unfavorable to the development of a true object-choice with its accompanying sexual overvaluation.” (Freud, 1914)

Thus, the difference between the girl's and the boy's position, already observed by Freud during the infantile period, reappears again after puberty. The boy's object choice is anaclitic with an overvaluation of the sexual object and can in fact turn into a paralyzing infatuation, akin to the courtly love of the Middle Ages, where the “object is elevated to the dignity of the Thing,” as Lacan puts it (Lacan, 2013). As for the girl, she sides with the Thing, presenting herself as inaccessible, taking a narcissistic position of seduction. She turns herself into an object impossible to grasp and thus provokes the boy's predatory greed. The arrival of a child, as Freud stresses, can later give her access to attachment-type love, similar to the choice made by the boy.

We understand that the girl's narcissistic position of seduction is a continuation of what she had already experienced toward the first man of her life, the father of early childhood—which is the opposite of what we see in the boy. For him, the new situation of puberty appears as a radical discontinuity. It corresponds to what we have described, in another work, as the *emergence of the Feminine* (Ouvry, 2001)—Feminine with a capital F, to mark its impossible inscription in what existed before its arrival, i.e., the infantile.

This emergence of the Other sex (again, for the same reasons, with a capital O), which did not exist in the infantile period [see Freud's discussion of the displacement, prior to puberty, of the erogenous zone from the clitoris to the vagina (Freud, 1933)] constitutes the second difference between the girl's and the boy's trajectories. This appearance is an effect of the Real, the “Real of puberty” (Ouvry, 1999), in other words an experience that cannot be inscribed in the infantile phallic organization, in language; it is a discovery of something radically new and unprecedented: The Feminine. This is what Lacan tries to express through his famous aphorism: “There is no sexual relationship”^5^. In other words, there is no relationship between the sexes in the structure of language, because there is only one signifier for both sexes: “There is no signifier of the Woman's sex.”

The boy's new position is therefore very far from where he stood before puberty. The world has been transformed, and before he can adjust this new reality to a figuration that is socially feasible (i.e., the other of the Other sex), there is a certain delay^6^. Time is needed to take on the social rules of relating to the other outside the incestuous framework of Oedipus (of the infantile). This intermediary period is also where we situate the phenomena of porn consumption and conspiracy thinking.

But before we discuss these phenomena, we would like to look at the logical consequences of what we have just said, specifically in relation to the girl's situation and trying to understand the higher prevalence of this behavior among boys.

For the girl, the emergence of the Real of puberty in fact happens at a later moment, when she discovers the Other jouissance of which she is the potential bearer. This discovery is made the first time she feels this jouissance during sex. The importance of this moment seems to be confirmed by a significant clinical fact, namely that certain young men can suffer a psychotic break when they first make a woman orgasm, due to the change in the woman's status. Until this moment, during the infantile period, it was the father who knew something about sexuality. And now it is the girl who finds herself the bearer of this knowledge, via her Other jouissance (effect of the Real of puberty). In psychosis, where the Name-of-the-Father has not been symbolized (foreclosure), the boy's summoning of the father of the infantile at this very moment necessarily has consequences. Contrary to the neurotic, where the effect is a certain deposition of the father (a fall of the great Other and the appearance of the S(A)), for the psychotic it is instead the hole of the Real that is suddenly revealed, setting off a cascade of effects that culminate in a delusional state (Lacan, 2006).

## Discussion

We have thus defined the two stages that characterize the first period post-puberty: for the boy, it is situated between the immediate experience of the Real of puberty and its “dressing up” by the other of the Other sex, which necessarily happens at a later stage; for the girl, it runs from the first physiological changes of puberty to the discovery of the Other jouissance, when she first has sex. When we visualize these two positions, we see that they are, just like for Freud during the infantile period, mirror images of one another: the woman's immediate and the man's delayed position of seduction; the experience of the Real of puberty, which is immediate for the boy and delayed for the girl.

Pornography and conspiracy thinking correspond to this intermediary phase^7^, specifically among boys. Both manifestations are de facto reactions to the Real of puberty, in terms of both its truth effect and the absence of knowledge linked to it (there is no signifier for the woman's sex). They can be related to each other using the following equation: pornography is to the sexual relationship what conspiracy thinking is to truth, or, *pornography is the conspiracy theory of the sexual non-rapport*^8^. What do we mean by this?

The effect of the Real of puberty bores a hole in the infantile knowledge guaranteed by the father. This is Lacan's *there is no sexual relationship* we are already familiar with. And it is indeed *knowledge* that conspiracy thinking wants to question^9^: “They are hiding something from us”, the argument goes, and these secrets belong to the domain of truth, in this case the truth of *not* knowing. However, what is the truth that puberty suddenly reveals, if not precisely the truth linked to jouissance—which, by definition escapes knowledge? Conspiracy thought is evidence of the adolescent's inevitable dilemma: “Should I accept the new situation of puberty, at the cost of revealing the non-knowledge that the Oedipal fiction helped conceal? Can I really leave the world of childhood so easily?”

On the other hand, pornographic images revive, as a kind of resurgence of the infantile, the knowledge about sexuality that was there to be had, the knowledge of the Oedipal promise, which is guaranteed by the father—the imaginary father of the infantile period, the father of the infantile phallic stage. In this case, this knowledge [*savoir*] is obtained by “seeing it [ç*a voir*]”—in other words, by seeing the sexual relationship as if it indeed existed, as if there really *was* something to see, even though it radically escapes representation, again and again^10^. In today's society, this strategy is greatly facilitated by the internet, which offers a whole new range of viewing possibilities^11^.

The idea that knowledge should somehow be preserved (that the infantile theory should be “saved,” like data on a disk) echoes the approach of the conspiracy thinker, his quest for a true knowledge that would give meaning to the official discourses, be they journalistic, historical and/or academic. These same discourses are in fact trying to circumvent the idea of a total knowledge, precisely by surfing on the non-knowledge they have embraced. They can dress up the current events or history or any other knowledge through a discourse of semblances, a semblant of truth that, as they know, by nature escapes them. In this way, they express what the other of the Other sex has come to conceal for them, namely the effect of the Real of puberty that we have discussed, which remains inaccessible to knowledge.

And this is what conspiracy thinking refuses and rejects, calling instead for a total knowledge that would be the truth. This “underground” knowledge could unmask the truth of the semblant, i.e., what psychoanalytic theory identifies as castration.

I will use a clinical vignette to illustrate my argument. It concerns a teenager with an interest in conspiracy theories, whose a family configuration where the mystic father blocker by a phallic mother has deprived him, since his childhood, from access to the symbolic phallic function, thus preventing him from discovering the Other jouissance of puberty. By resorting to watching porn videos, he is able to maintain an infantile auto-erotic sexuality through masturbation, while women in fact remain a source of dread.

## Concluding remarks

With “seeing it” [ç*a voir*] and knowledge [*savoir*], we have two valences of the same principle: applied to sexuality (pornography) and to truth (conspiracy theories). Both attest to the same refusal of the emergence of the Other jouissance, the same attempt to avoid non-knowledge, non-sense, non-truth (the truth is a lying truth, Lacan said)—an attitude that in fact assumes a destitution of being^12^, of the being of truth that is the father of the infantile period.

Both these valences must be transitory, marking simply a stage in the complicated passage of the boy—and of some girls, who assume the masculine sexual position. Only clinical analysis can identify situations where there is a true risk of pathological fixation. At a time of hardening attitudes and growing fears (which after the attacks of 2015 are of course legitimate), we must remain even more rigorous in our clinical approach, which alone can help us contain our emotions and not become overwhelmed by them, a risk that easily leads to premature warnings and false alarms.

## Author contributions

The author confirms being the sole contributor of this work and approved it for publication.

### Conflict of interest statement

The author declares that the research was conducted in the absence of any commercial or financial relationships that could be construed as a potential conflict of interest.

## Footnotes

^1^A conspiracy theory is an explanation of an event or situation that invokes an unwarranted conspiracy, generally one involving an illegal or harmful act carried out by government or other powerful actors. Conspiracy theories often produce hypotheses that contradict the prevailing understanding of history or simple facts. The term is a derogatory one (Ayto, 1999). According to the political scientist Barkun, conspiracy theories rely on the view that the universe is governed by design, and embody three principles: nothing happens by accident, nothing is as it seems, and everything is connected (Barkun, 2003). Another common feature is that conspiracy theories evolve to incorporate whatever evidence exists against them, so that they become, as Barkun writes, a closed system that is unfalsifiable, and therefore “a matter of faith rather than proof” (Barkun, 2003, 2011).

^2^A new work brings a point of view different from ours as for the adolescence and the pornography. It is complementary in the fact that he proposes the festive point of view of the use of the porn (Bidaud, 2017).

^3^The judicious article of Janin (2015) addresses this issue from a complementary angle, namely shame, hatred and pornography, whereby the first two terms can be related to the rapport of the Other sex.

^4^We should stress, as Freud also does, that the girl's trajectory through the pre-Oedipal stage and then through puberty can only be tracked via the differences from the boy's position. Freud writes: “In conformity with its peculiar nature, psycho-analysis does not try to describe what a woman is—that would be a task it could scarcely perform—but sets about enquiring how she comes into being, how a woman develops out of a child with a bisexual disposition.” (Freud, 1933).

^5^This aligns with Freud's lecture on *Femininity* (see Note 4). In the same text, he also stresses that there is no specifically feminine libido, which is another way of putting of what he has said previously, namely that nothing in language could express what a woman is, and thus we have no means to describe a feminine trajectory or feminine libido. His words are later echoed by Lacan's formulations *(the Woman does not exist*, or *no signifier exists for a woman's sex)—*or rather, to be precise, Lacan's commentary renders Freud's argument more legible.

^6^Cf. Choudhury et al. (2006) “Changes in social behavior are driven by both social and biological factors. During adolescence, it is likely that peer interactions and societal influences as well as genetically determined hormonal milieu influence social behavior. However, since the recent discovery that the brain matures considerably during adolescence, evidence has emerged pointing to the role of neural maturation in the development of social cognition during adolescence.”

^7^Cf. Marty F., La violence comme expression du mal-être à l'adolescence, *Adolescence* 2009/4 (n° 70), 1007–1017.

^8^Rob Brotherton, Suspicious Minds, Bloomsbury Sigma, 2017-01-03, 304 pages, ISBN-10: 1472915631, quoted by Lynne alcolm, http://www.abc.net.au/radionational/programs/allinthemind/the-psychology-of-conspiracy-theories/7177962,17 February 2016: “This is something that we all suffer from, not just in the context of conspiracy theories, but we all want to feel like we have control over our circumstances and that we understand what's going on around us. When that feeling of control is stripped away for whatever reason…then we look for other sources of control, what is called compensatory control.”

^9^Cf. Sander van der Linden (London School of Economics and Political Science, Grantham Research Institute on Climate Change and the Environment), What a hoax, *Scientific American Mind 41*, September/October 2013 (Mind.ScientificAmerican.com): “A strong distrust of authority would be one such overarching ideological lens. In a belief system in which authorities are fundamentally untrustworthy, alternative—even outlandish and contradictory—explanations for troubling events can seem plausible, as long as they are consistent with a skepticism toward the powers that be.”

^10^Like those who engage in watching pornography and always repeat the same scene—desperately fleeting, desperately empty.

^11^There is of course nothing new about this phenomenon: just think of Antoine Doisnell in *400 Blows* (Truffaut, 1959), when he and his friend are looking at the photographs in mail-order catalogues, where scantily-clad women become the subject of laughs, looks, research and frustration.

^12^Lacan, J. (1973–1974). *Les non-dupes errent* (Unpublished): “Real is the reason for the lying truth. If only because it is variable. What do we call the Real? It is what cannot be said otherwise than in lying, what is reluctant to the truth, reluctant to say that it is true.
